# Mechanisms of induction of regulatory B cells in the tumour microenvironment and their contribution to immunosuppression and pro-tumour responses

**DOI:** 10.1093/cei/uxac029

**Published:** 2022-03-29

**Authors:** Fabian Flores-Borja, Paul Blair

**Affiliations:** Centre for Oral Immunobiology and Regenerative Medicine, Barts & The London School of Medicine and Dentistry, Queen Mary University of London, Blizard Institute, 4 Newark Street, London E1 2AT, UK; Division of Infection & Immunity, Faculty of Medical Sciences, Department of Infection, Immunity, and Transplantation, University College London. The Pears Building, Rowland Hill St, London NW3 2PP, UK

**Keywords:** regulatory B cells, tumour immunology, IL-10, regulatory T cells

## Abstract

The presence of tumour-infiltrating immune cells was originally associated with the induction of anti-tumour responses and good a prognosis. A more refined characterization of the tumour microenvironment has challenged this original idea and evidence now exists pointing to a critical role for immune cells in the modulation of anti-tumour responses and the induction of a tolerant pro-tumour environment. The coordinated action of diverse immunosuppressive populations, both innate and adaptive, shapes a variety of pro-tumour responses leading to tumour progression and metastasis. Regulatory B cells have emerged as critical modulators and suppressors of anti-tumour responses. As reported in autoimmunity and infection studies, Bregs are a heterogeneous population with diverse phenotypes and different mechanisms of action. Here we review recent studies on Bregs from animal models and patients, covering a variety of types of cancer. We describe the heterogeneity of Bregs, the cellular interactions they make with other immune cells and the tumour itself, and their mechanism of suppression that enables tumour escape. We also discuss the potential therapeutic tools that may inhibit Bregs function and promote anti-tumour responses.

## Teaser

This article describes the induction of regulatory B cells in the tumour microenvironment. We discuss different phenotypes of Bregs and their mechanisms of suppression of anti-tumour responses. We also discuss the therapeutic implications of inhibition of Bregs in cancer.

## Introduction

Regulatory B cells were first described in the context of autoimmunity and are now recognized to play important roles in infection, allergy, and tolerance to transplants [1, 2]. Bregs have also been characterized in the oncology arena, and an important focus of research is how Bregs, or their precursors, develop and are recruited to the tumour microenvironment [3], and what cellular and molecular mechanisms are involved in their suppression of anti-tumour immunity [4].

The fact that immune cells infiltrate tumours has been recognized for a long time. The vast majority of tumour-infiltrating cells comprise cytotoxic CD8^+^ and helper CD4^+^ T cells, but also include a minority of B lymphocytes and natural killer cells [5, 6]. Pioneering studies suggested that these cells had a key role in the modulation of the immune responses against tumours and are associated with better prognosis [7]. However, it has now been established that certain immune cells can also induce pro-tumour effects associated with negative clinical outcomes. Basic and clinical research has identified regulatory T cells (Tregs) [8], myeloid-derived suppressor cells (MDSCs) [9], and tumour-associated macrophages (TAMs) amongst the cells that orchestrate the induction of an immunosuppressive environment that promotes tumour development, evasion, progression, and metastasis [10]. A more refined characterization of the TME has also identified B cells as key players in the modulation of the immune response in cancer [11]. B cells infiltrate tumours and can display opposing functions that have important effects on tumour rejection or progression and metastasis. In some tumours, B cells represent a considerable portion of infiltrating cells and contribute to tumour rejection by presenting antigens, by secreting tumour-targeting antibodies that induce tumour cell apoptosis, and by secreting cytokines that prime effector CD4^+^ and cytotoxic CD8^+^T cells [12, 13]. There are, however, subsets of Bregs that contribute to tumour progression due to their ability to induce a tolerogenic environment with increased numbers and activity of immunosuppressive cells [14]. Just as their counterparts described in autoimmunity, infection, allergy, or transplantation, many different populations of Bregs with immunosuppressive activity have been described in the TME. The lack of a Breg-specific marker (like Foxp3 for Tregs) presents a challenge in the field making their identification in different settings inconsistent. Not all previously defined Breg populations have been described in human or mouse cancers, and the populations described in cancer do not always appear to be equivalent to those in health or autoimmunity (see Table 1). For some degree of consistency, Bregs can be defined functionally based on their ability to produce suppressive and anti-inflammatory cytokines (IL-10, TGFβ, and IL-35) that suppress pro-inflammatory or cytotoxic anti-tumour cells, and we have used this format to describe Bregs in a cancer setting. In this review, we will focus on the mechanisms leading to the induction of Bregs and the mechanisms and functional responses that allow them to suppress anti-tumour responses.

**Table 1:** Phenotype and mechanism of action of major populations of Bregs identified in autoimmune conditions and different types of human and mouse cancer. EAE, experimental autoimmune encephalomyelitis; SLE, systemic lupus erythematosus.

Phenotype	Disease model	Mechanism	References
Mouse			
CD19^+^CD5^+^CD1d^hi^ (B10)	Health, EAE	IL-10-mediated suppression	[15]
CD19^+^CD21^+^CD23^+^CD24^+^CD1d^+^ (T2-Bregs)	Arthritis		[16]
CD19^+^TIM-1^+^ (TIM-1 Bregs)	Transplant tolerance		[17]
LAG3^+^CD138^hi^Blimp1^hi^CD1d^hi^CD200^hi^PDL1^+^PDL2^+^ (LAG3^+^ Plasma cells)	EAE	IL-10- and IL-35-mediated suppression	[18]
Human			
CD19^+^CD24^hi^CD38^hi^CD1d^hi^	SLE	IL-10-medited suppression	[19]
CD19^+^CD24^hi^CD27^+^	Rheumatologic disorders		[20]
TIM-1^+^ Bregs	Systemic sclerosis		[21]
Mouse CD19^+^B220^+^CD25^+^CD69^+^	Breast cancer	TGFβ-mediated induction of Tregs; decreased ratio of Th1/Th17	[22]
Human CD19^+^CD25^+^IL-10^+^	Breast cancer	IL-10-mediated suppression	[23]
Human CD19^+^IL-10^+^	Cervical and ovarian cancer, tongue squamous cell carcinoma	Induction of Tregs	[24–26]
Human CD19^+^CD24^+^CD38^+^	Breast cancer	Induction of Tregs	[27, 28]
	Acute myeloid leukaemia	IL-10-mediated suppression	[29]
	Hepatocellular carcinoma	IL-10- and IL-35-mediated suppression mechanisms	[30]
		TGFβ and CD40/CD154 pathway-mediated tumour promotion	[31]
Human CD19^+^CD24^hi^CD27^+^	Squamous cell carcinoma	IL-10-mediated suppression	[32]
Mouse CD19^+^IL-10^+^PD-1^+^	Hepatocellular carcinoma	IL-10-mediated inhibition of NK and CD8^+^T cells	[33]
Human CD5^hi^CD24^-/+^ CD27^hi/+^CD38^dim^ PD-1^hi^	Hepatocellular carcinoma	IL-10-mediated suppression	[34]
Human CD19^+^PD-1^hi^	Squamous cell carcinoma	IL-10-mediated suppression	[32]
Human CD19^+^CD24^hi^CD27^+^	Gastric cancer	Suppression of IFNγ secretion by CD4^+^ T cells	[35]
Human CD5^high^CD24^-^CD27^−/+^CD38^+/high^ TIM-1^+^	Hepatocellular carcinoma	IL-10 mediated suppression of CD8^+^ T cells	[36]
Human CD19^lo^CD27^hi^TIM-1^hi^	Colorectal cancer	Suppression of IFNγ and TNFα secretion	[37]
Human CD19^+^PD-1^+^PD-L1^+^	Thyroid cancer	PD-L1-mediated suppression of CD4^+^ and CD8^+^ T cells	[38]
Human and mouse CD20^+^PD-L1^+^	Glioblastoma	IL-10 and TGFβ- mediated suppression of CD8+ T cells	[9]
Human and mouse CD19^+^PD-1^-^PD-L1^+^	Breast cancer	PD-L1-mediated suppression	[39]
Mouse CD25^+^CD86^+^PD-L1^+^ PD-L1^hi^	Breast cancer	PD-L1 and TGF-β mediated suppression of T cells proliferation and cytokine secretion	[40]
Mouse CD19^+^CD81^+^CD27^+^CD25^+^PD-L1^hi^	Fibrosarcoma	TGF-β-mediated inhibition of T cells activation	[41]
Human and mouse CD19^+^B220^lo^CD138^+^IgA^+^	Hepatocellular carcinoma	PD-L1-mediated suppression	[42]
Mouse CD19^+^IL-10^+^PD-L1^+^IgA^+^	Colorectal and prostate cancer	Suppression of CD8^+^ cytotoxic cells; lymphotoxin-mediated induction of tumour progression	[43, 44]
Mouse CD19^+^CD1^hi^CD5^+^	Lymphoma,	IL-10-mediated inhibition of ADCC by monocytes	[45]
	Pancreatic cancer	IL-35-mediated suppression and induction of tumour growth	[46, 47]
Mouse CD19^+^CD5^+^CD43^+^	Pancreatic cancer	PD-L1-mediated suppression of CD8^+^ T cells	[48]
	Melanoma	IL-10 mediated suppression IFNγ and TNFα by CD8^+^ T cells	[49]
Mouse CD19^+^CD5^-^	Melanoma	Tumour progression via STAT3-regulated angiogenesis	[50, 51]
Human CD38^+^CD1d^+^IgM^+^CD147^+^	Mammary, ovarian, cervical, colorectal and prostate carcinoma	Granzyme B-mediated inhibition of T cells proliferation	[52]
Mouse CD19^+^CD21^high^	Skin carcinoma	TNFα−mediated induction of papilloma development	[53]
Human CD20^+^CD27^-^	Melanoma	PD-L1-mediated suppression of T cells	[54]
Human CD19^+^CD49b^+^CD73^+^IgG4^+^	Melanoma	Secretion of pro-angiogenic factors	[55]

## Mechanisms of induction of tumour-associated Bregs

### Tumour-derived factors

Tumour cells exploit the mechanisms of immune tolerance by inducing or recruiting populations of immunosuppressive cells that deploy inhibitory mechanisms allowing tumour progression. One of the first reports on the presence of tumour-induced Bregs came from studies in a mouse model of breast cancer. A B-cell subset expressing activation markers (CD25^+^CD69^+^B7-H1^+^CD81^+^CD86^+^) and termed tumour-evoked Bregs (tBreg) induced the generation of Foxp3^+^Tregs, which inhibited anti-tumour responses [22]. The induction of Tregs by tBregs in this model was not mediated by IL-10, but by TGFβ. The important role of these cells and their contribution to metastasis to the lungs was demonstrated by selective targeting and depletion of this subset with anti-CD25 or B220 antibodies. Treatment with these antibodies resulted in abrogation of lung metastasis. In this model, it was shown that stimulation of B cells with breast cancer cell-conditioned medium induced the development of Bregs. However, the identity of the factor(s) responsible was not determined in this study [22]. Further characterization showed that this subset can be induced by leukotriene B_4_ (LTB_4_), a lipid metabolite of the 5-lipoxigenase signalling pathway produced by breast cancer cells. LTB_4_ binds peroxisome proliferator activator-receptor α (PPARα) expressed by B cells and induces the generation of tBregs which facilitate tumour escape [56].

Tumour cells can also induce Bregs by direct cell contact and cytokine production. The contribution of the PD-L1/PD-1 signalling axis to the development and suppressive function of Bregs has been highlighted in recent studies. Culture of PD-L1 expressing human epithelial breast cancer cells with B cells can induces the development of CD24^+^CD38^+^Bregs, which are associated with increased tumour grade and higher frequency of Tregs in patients with invasive breast cancer [27]. Analyses of mouse models and samples from patients with pancreatic cancer have shown that IL-18-producing tumour cells induce the development of PD-1-expressing Bregs. These suppressor cells inhibit cytotoxic T and NK cells through IL-10 secretion [57].

**Figure 1:**
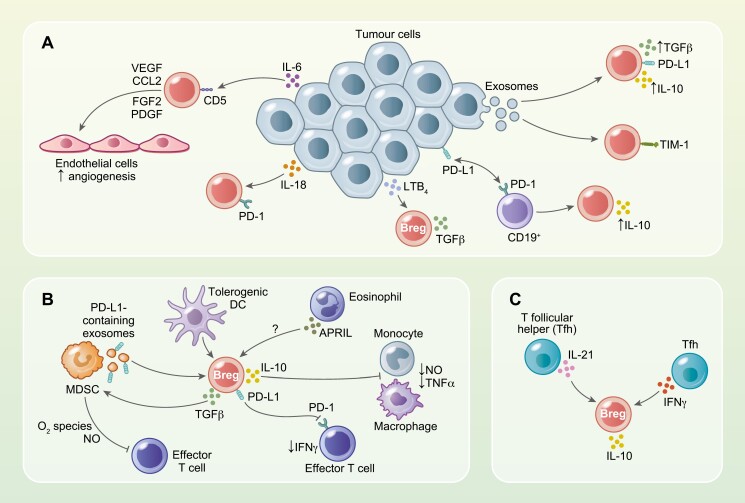
Cellular and molecular mediators of Breg induction in the tumour microenvironment. Bregs in the tumour microenvironment are induced by mechanisms involving molecules expressed or secreted by tumour cells. (**a**) Tumour-derived exosomes induce the generation of PD-L1- and TIM-1-expressing Bregs that produce IL-10 and/or TGFβ. PD-L1-expressing tumour cells induce the conversion of effector B cells into IL-10-producing Bregs. Lipid metabolites such as leukotriene B4 induce TGFβ-producing Bregs. Tumour-derived cytokines such as IL-18 induce suppressive PD-1-expressing Bregs, whilst IL-6 induces the production of endothelial growth factors by CD5^+^ Bregs that contribute to angiogenesis. (**b**) Innate immune cells such as myeloid-derived suppressor cells (MDSCs) and tolerogenic dendritic contribute to the generation of IL-10-producing and PD-L1-expressive Bregs. These cells can downregulate the cytotoxic activity of monocytes and macrophages. PD-L1-containing exosomes released by MDSCs induce Breg to secretion of TGFβ, which in turn activates MDSC to produce oxygen reactive species and nitric oxide (NO) to inhibit effector T cells. (c) IL-21 and IFNγ-producing T follicular helper cells are adaptive immune cells that can induce IL-10-producing Bregs.

Tumour cells, and some TME resident immune cells, also secrete exosomes that mediate the generation of IL-10 producing and PD-1-expressing Bregs. Exosomes, which are small cell-derived vesicles of 30–100 nm diameter, contain mRNAs and microRNAs that are translated to proteins by, and/or regulate gene expression in, cells that take them up [58]. Mao *et al*. demonstrated that exosomes derived from patients with oesophageal squamous cell carcinoma contain mRNAs associated with TLR4 and MAPK signalling that induce the generation of IL-10^+^ and PD1^+^ Bregs from healthy peripheral blood CD19^+^ B cells [32]. Microvesicles, 100–1000 nm cell derived particles that form from blebbing of the plasma membrane [59], can also generate Bregs in cancer. Lee-Chang et al. showed that glioblastoma-infiltrating MDSCs secrete PD-L1-containing microvesicles that are taken up by tumoral B cells. The acquisition of membrane-bound PD-L1 confers a suppressive phenotype to these B cells which subsequently acquire the ability to express TGFβ and IL-10 (**10**). Another exosome-mediated mechanism that generates Bregs has been unveiled in liver cancer. High mobility group box 1 protein (HMGB1) present in exosomes derived from hepatocarcinoma cells induces a population of IL-10-secreting TIM-1^+^Bregs (CD5^high^CD24^-^CD27^−/+^CD38^+/high^) which is associated with advanced stages of cancer development and reduced patient survival [36]. These studies suggest that tumour-derived factors regulate the differentiation of suppressive immune cell types that promote tumour survival, progression, and metastasis (summarized in Fig. 1a).

### Innate immune cells

Innate immune cells can also be found in the TME and have been suggested to induce Breg differentiation in both mice and humans. Characterization of the immune components in the 4T1 breast cancer model revealed that cell-to-cell interactions between MDSCs (CD11b^+^Gr-1^+^) and B cells alter B cell functions (proliferation, apoptosis, and secretion of cytokines and antibodies) and expression of checkpoint-associated molecules PD-1 and PD-L1. In tumour-bearing mice, MDSCs induce the emergence of a population of splenic PD1^-^PD-L1^+^CD19^+^IL-10-secreting B cells that inhibit effector T cell proliferation and IFNγ expression [39]. The effects of the interactions between MDSCs and Bregs are in fact in both directions as tBregs can increase the suppressive function of monocytic and granulocytic MDSCs. TGFβ produced by Bregs can increase the ability of TGFβR1 and TGFβR2 expressing MDSCs to produce cytotoxic reactive oxygen species and nitric oxide (NO), resulting in efficient inhibition of CD4^+^ and CD8^+^T cell anti-tumour responses [60]. In addition to activating suppressive innate cell function Bregs can inhibit their anti-tumour activity. For instance, CD1d^hi^CD5^+^ Bregs produce IL-10 and downregulate the production of NO, TNFα, and the expression of activation markers by monocytes and macrophages limiting their anti-tumour activity. This phenomenon is of therapeutic importance as even small numbers of CD1d^hi^CD5^+^Bregs can abrogate the antibody-mediated depletion of lymphoma cells, an anti-tumour response that depends on the cytotoxic activity of macrophages and monocytes [45]. Dendritic cells (DCs) are another subset of innate immune cells with the potential of inducing Bregs. Due to their ability to present antigens, DCs in the TME contribute to anti-tumour responses by activating effector T cells. However, under certain circumstances, the TME induces DC polarization and the adoption of a tolerogenic profile that contributes to the generation of suppressor cells allowing tumour progression [61]. *In vitro* analyses have shown that stimulation with dexamethasone and 1α,25-dihydroxyvitamin D3 produces tolerogenic DCs that can generate Tr1 (CD4^+^IL-10^+^CD25^-^Foxp3^-^) and IL-10^+^Bregs from healthy PBMCs [62]. Further, in models of autoimmunity it has been reported that IgA-producing Bregs are generated under the influence of A proliferation-inducing ligand (APRIL). Like their autoimmune counterparts, IgA^+^ Bregs in cancer suppress T cells and macrophages by IL-10- and PD-L1-mediated mechanisms [43, 63]. A recent study has shown that eosinophils are a source of APRIL, a cytokine that drives the generation of Bregs in the context of gastric lymphoma [64]. This suggests that eosinophils, which have been detected in the TME in several types of cancer [65], could also play an important role in the generation of Bregs in cancer (Fig. 1b summarizes potential innate cell contribution to Breg generation in cancer).

### Adaptive immune cells

Adaptive immune cells have also been shown to contribute to Breg development. In non-small cell lung cancer (NSCLC), functionally deficient T follicular helper cells (Tfh), with impaired ability to secrete IL-21, can induce Bregs differentiation. Culturing PD-1^+^Tfh with PD-L1-expressing NSCLC cells preferentially expands IL-21-deficient Tfh which can then expand IL-10^+^Bregs [66]. Another subset of Tfh, termed T follicular regulatory cells, induces the differentiation of IL-10-producing B cells in breast cancer [67] (Fig. 1c). In contrast, some Tfh subsets can inhibit Breg generation in cancer. For instance, a population of IFNγ secreting Tfh (Tfh1) inhibits the development of CD24^+^CD38^+^ Bregs in *Helicobacter pylori (H. pylori)*-infected gastric cancer patients. Surprisingly, this is associated with a worse outcome as *H. pylori*-infected patients with high levels of Bregs did not develop gastric cancer. These results suggest that Bregs might downregulate the tissue-damaging pro-inflammatory responses triggered by bacterial infection that generate an environment that favours tumour progression [68]. These observations and the fact that several types of cancer are associated with chronic inflammation due to infection add another level of complexity to the biology of Bregs in cancer, especially as recent studies have shown the important role of the intestinal microbiome in the induction of Bregs [69, 70].

## Bregs mechanisms for tumour promotion and suppression of anti-tumour responses

### IL-10

In the majority of studies of Breg biology across all fields, IL-10 expression has been suggested to be their primary mechanism of suppression. IL-10^+^ Bregs and their positive correlation with Tregs [23], suppression of CD4^+^ and CD8^+^T cells anti-tumour responses, and their induction of a tolerant TME have now been described in numerous solid and haematological cancers including liver, cervical, ovarian, gastric, and breast cancers, head and neck squamous cell carcinoma (HNSCC) [23–26, 29, 35], acute myeloid leukaemia, and non-Hodgkin lymphoma [8, 30, 71–73]. However recent studies have shown that a variety of B cell subpopulations display IL-10-dependent suppressive immune regulatory functions [11, 13, 74]. For instance, CD19^lo^CD27^hi^B cells detected in colorectal cancer and expressing high levels of TIM-1 have potent IL-10-mediated immunosuppressive function [37]. Studies with TIM-1-expressing Bregs have provided insight into the intracellular molecular mechanisms that control IL-10 production and suppressive function of Bregs. TIM-1^+^B cells in leukaemia have decreased levels of microRNAs MiR-15a/16-1 which targets the 3ʹ-untranslating region of STAT3. With decreased MiR-15a/16-1, STAT3 levels and activity increase resulting in IL-10 production [75]. Similarly, B1a Bregs (CD19^+^CD5^+^CD43^+^) have been described to use IL-10 to inhibit IFNγ and TNFα production by CD8^+^T cells thus negatively regulating tumour immunity against melanoma cells [49].

**Figure 2:**
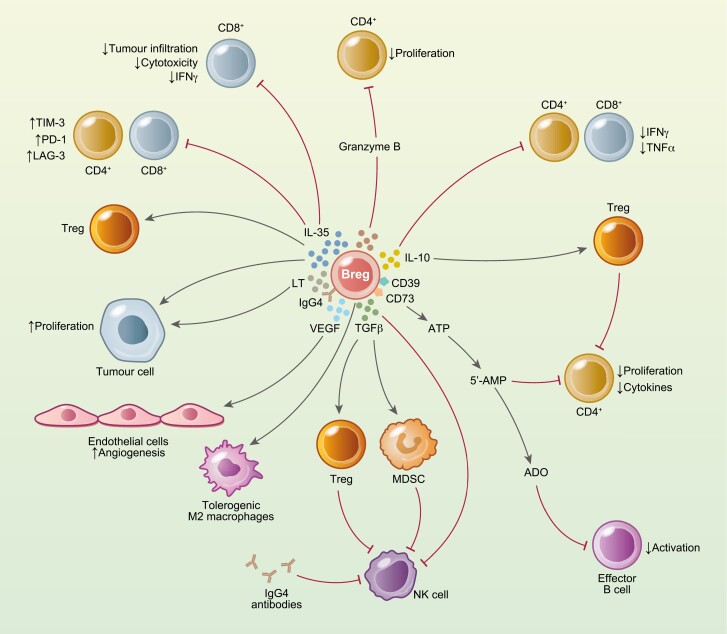
Mechanisms of suppression by Bregs in the tumour microenvironment. Different populations of Bregs produce IL-10, IL-35, and TGFβ that mediate the suppression of anti-tumour responses by T cells. All three regulatory factors also induce the generation of Tregs. IL-10 and IL-35 suppress tumour infiltration, proliferation, cytokine production, and cytotoxic activity of CD4^+^ and CD8^+^T cells. IL-35 and lymphotoxin (LT) can act as growth factors for tumour cells. VEGF produced by Bregs can act on endothelial cells and promote angiogenesis. In addition, IL-35 induces the expression of negative immune checkpoint molecules TIM-3, PD-1, and LAG-3. A population of Bregs secretes granzyme B that inhibits the proliferation of CD4^+^ T cells. Bregs can also express ectonucleotidases CD39 and CD73 that convert ATP into 5ʹAMP and ADO to inhibit CD4^+^ T cells and effector B cells, respectively. TGFβ produced by Bregs can directly, or indirectly through the induction of MDSCs and Tregs, downregulate NK cell activity. IgG4^+^ B cells induce tolerogenic M2-like macrophages and inhibit ADCC by binding FcγRI.

However, as in the fields of autoimmunity and infection, it is evident that in the context of cancer there is also a diversity of Bregs and many of these Bregs may deploy IL-10-independent suppressive mechanisms as well as, or instead of, IL-10 expression [76]. A summary of these mechanisms is shown in Fig. 2. For instance, in hepatocellular carcinoma, while there are increased numbers of IL-10^+^ Bregs in the peripheral blood, which are indicative of the generation of a pro-tumour environment, there are also increased levels of IL-35, a cytokine that has recently been associated with Breg function [30]. A second study of hepatocellular carcinoma also implicated TGFβ in Breg suppressive function. Here, CD24^+^CD38^+^ Bregs promote tumour progression through a mechanism involving CD40 and CD40L interactions with cancer cells that subsequently leads to the secretion of IL-10 and TGFβ that sustain tumour proliferation [31]. In the next subsection, we will describe several IL-10-independent mechanisms of suppression mediated by Bregs in the TME.

### IL-35

IL-35-producing B cells have recently been identified as key negative regulators of immunity. With a phenotype resembling plasma cells (IgM^+^CD138^hi^CD19^+^) this subset of Bregs function as APCs and modulates the activation of macrophages and T cells in models of bacterial infection [18, 77]. In pancreatic and gastric cancer, different populations of IL-35-secreting Bregs display suppressive mechanisms that go beyond the generation of Tregs [46, 78]. Mirlekar et al. showed that IL-35, specifically produced by Bregs, triggers activation of transcription activator STAT3 in gp130^+^CD8^+^ T cells leading to inhibition of chemotactic receptors (CXCR3 and CCR5) and IFNγ expression. This results in blocking tumour infiltration and inhibition of cytotoxic function by CD8^+^T cells [79]. Another pro-tumour effect of B cell produced IL-35 has been described in gastric cancer. In that study increased levels of IL-35^+^B cells were found in advanced stages of cancer, and this was associated with increased frequency of Tregs [80]. In a model of pancreatitis and liver metastasis, the crucial role of B cells was demonstrated when antibody-mediated depletion of B cells resulted in diminished pancreatic intraepithelial neoplasia. Refined analyses showed that Breg-derived IL-35 induced the expression of PD-L1 in B cells which then suppressed CD8^+^ T cells [48]. Studies with pancreatic tumour cells suggested that in some settings B cell-derived IL-35 drives tumour growth not only by downregulating anti-tumour responses but also by acting as an endogenous growth factor that promotes tumour cell proliferation and inhibits apoptosis [81]. Indeed, 2 years later direct evidence was obtained showing that IL-35 produced by CD1d^hi^CD5^+^ Bregs acted as a growth factor for tumour cells [47]. Similar to B-cell IL-35 production, IL-35 produced by Tregs in the TME induces a state of cell exhaustion and dysfunction of CD4^+^ and CD8^+^ T cells characterized by increased expression of negative immune checkpoints TIM-3, PD-1, and LAG3 [82]. In addition, tumour cells themselves can produce IL-35 and support the generation of Tregs and recruitment of CD11b^+^Gr-1^+^MDSCs [83, 84] that contribute to angiogenesis and pro-tumour activities. All these studies together suggest that cooperative IL-35 mediated interactions might be established between MDSCs, Bregs, and Tregs to generate a TME that promotes cancer progression.

### TGFβ and pro-inflammatory cytokines

Early studies in non-Hodgkin lymphoma showed that suppressive B cells expressed membrane-bound TGFβ that promoted the expression of Foxp3 and the development of Tregs, suggesting that Bregs could be pro-tumorigenic in cancer [73, 85]. More recent work has directly proved a suppressive role for TGFβ expression by Bregs in the inhibition of cancer immune responses. For instance, inhibition of the TGF receptor (TGFR) with small-molecule inhibitors abolishes the suppression established by CD81^+^CD27^+^PD-L1^+^B cells in a model of fibrosarcoma [41]. Interestingly, here the authors reported that tBregs expressed constitutively active STAT3, and future experiments proved that this signalling protein is crucial for the TGFβ suppressive phenotype in Bregs. Studies with polyphenol molecule resveratrol, which specifically inactivates STAT3, show that it inhibits the development of Bregs and TGFβexpression, resulting in restoration of anti-tumour immune response and control of inhibition of tumour growth by effector T cells [9]. The expression of membrane-bound TGF-β and PD-L1 confers on Bregs the ability to control NK cell activity and to impair anti-tumour responses in mouse models of breast cancer [40]. As reported for Tregs, TGFβ expressed by Bregs can downregulate the expression of the activating receptor NKG2D by NK cells and by doing this inhibit NK cell function [86]. In another IL-10-independent suppression mechanism, Bregs can induce the recruitment of Tregs to mouse mammary primary tumours which are associated with a decreased NK cell activity [76]. Further, by supporting the expansion of suppressive Tregs and MDSCs, Bregs can also downmodulate indirectly anti-tumour NK cell activity [74, 87] (see Fig. 2). Further characterization of TGFβ-producing Bregs showed that this subset can also express PD-L1 (the function of which is discussed below) [40].

Bregs whose action is IL-10-independent also includes TNFα-producing B cells that decrease the frequency of cytotoxic CD8^+^ T cells and promote papilloma development in a model of chemically induced skin carcinoma [53]. Another mechanism by which B cells promote tumour progression has been described in castration-resistant prostate cancer where B-cell lymphotoxin expression has been implicated. In this model, B cells were found to be recruited to primary tumours in a CXCL13-dependent fashion. There they produced lymphotoxin which contributed to the activation of prostate tumour cells IKKα and STAT3, resulting in androgen-independent growth of prostate cell tumours [88]. Further analyses determined that immunosuppressive B cells had an IgA^+^IL-10^+^PD-L1^+^ plasmocyte phenotype [44]. Granzyme-B expression by Bregs has also been reported to contribute to cancer immune evasion. Lindner *et al.* described that granzyme-B expressing B cells could be detected in sections from multiple solid-organ cancers, including mammary, cervical, ovarian, colorectal, and prostate carcinomas. These Bregs had a CD38^+^CD1d^+^IgM^+^CD147^+^ phenotype and were induced by IL-21 secreting regulatory T cells (IL-21 expression also being detected within the tumour tissue). These Bregs were subsequently able to inhibit the proliferation of T cells by a GrB-dependent degradation of the TCRζ chain. This suppressive mechanism is also used by Tregs, so it is possible that this subset of Bregs cooperates with Tregs to suppress anti-tumour responses mediated by T cells [52].

### PD-1/ PDL1

The PD-1/PD-L1 axis not only contributes to the generation of Bregs (as mentioned above) but is also part of the suppressive mechanisms used by Bregs in the TME. Xiao et al. identified PD-1^+^ tumour-infiltrating Bregs in hepatocellular carcinoma that contributes to tumour progression. This subset is distinct from peripheral CD24^+^CD38^+^Bregs and presents a CD5^hi^CD24^−/+^CD27^hi/+^CD38^dim^ phenotype, produces IL-10 and its development depends on TLR4-mediated BCL6 upregulation [34]. PD-L1 expressing Breg subsets (CD20^+^CD27^−^ Bregs) have also been described to suppress T-cell-mediated anti-tumour response in melanoma patients [54].

### Angiogenesis

Angiogenesis, epithelial cell proliferation, and the recruitment of innate immune cells that contribute to chronic inflammation in the TME are pro-tumour mechanisms that can be modulated by B cells [89]. Evidence from different types of cancer suggests that B cells can have a pro-angiogenic capacity and the ability to modulate endothelial cell function. In tumour samples from patients with melanoma and prostate cancer, the mechanism involves the activation of intrinsic STAT3 in B cells leading to the secretion of angiogenic factors S1PR1, MMP9, HIF1a, VEGF, and their accumulation around micro vessels in the tumour [50]. Further evidence of a role for Bregs in the promotion of angiogenesis was provided by Zhang *et al*. Here it was found that CD5^+^B cells are activated by direct ligation of CD5 by IL-6 leading to the upregulation of STAT3 by CD5^+^B cells. This leads to the secretion of the pro-angiogenic factors VEGF and CCL2 by the B cells which support tumour vascularization [51]. Evidence for the pro-angiogenic role of B cells has also been provided by transcriptomic analysis of immortalized B cells from the peripheral blood. Van de Veen *et al.* identified a population of CD49b^+^CD73^+^IgG4^+^B cells that produce VEGF, CYR61, AIDM, FGF2, PDGFA, and MDK that contribute to the activation of an “angiogenic switch” that favours metastasis in several types of cancer. Indeed, an increased frequency of peripheral and infiltrating CD49b^+^CD73^+^B cells was found in peripheral blood and tumour lesions of patients with melanoma, a malignancy characterized by its high metastatic potential [55]. *In vitro* functional assays showed that supernatants derived from BCR-activated CD49b^+^CD73^+^B cells induced the formation of endothelial cell tubes, strengthening the evidence that Bregs contribute to angiogenesis. Put together, these data point to B cells being major players in a sophisticated and highly orchestrated set of processes controlling tumour angiogenesis.

### CD39 and CD73

Another mechanism of suppression of T cell anti-tumour responses is mediated by Bregs expressing CD39 and CD73 [90]. These two ectonucleotidases catalyze the conversion of extracellular ATP into 5ʹ-AMP and adenosine, which are recognized by adenosine receptors expressed by several cell types, including Tregs and APCs. *In vitro* studies showed that activated B cells produced 5ʹ-AMP which upon recognition by adenosine receptor A3-R inhibited cytokine production and proliferation of effector T cells [90]. The metabolic conversion of ATP and binding to A1-R has been recognized as one of the main suppressive mechanisms deployed by Tregs [91, 92]. In the context of cancer, the major consequence of adenosine production is the induction of an immunosuppressive environment characterized by the generation of Tregs with increased functional activity, the downregulation of effector T cell function (increased apoptosis and anergy phenotype) and the induction tolerogenic APCs [93, 94]. The co-existence of adenosine-producing B cells and Tregs in the tumour microenvironment suggests a cooperative effect between these two suppressive lymphocyte subsets that might predict a negative outcome for cancer patients. A study of HNSCC cancer demonstrated that Breg adenosine production may also have consequences for the function of effector B cells. Adenosine produced by CD39^+^CD73^+^Bregs was recognized by effector B cells leading to inhibition of intracellular Ca^2+^ release and BTK signalling. By inhibiting B effector cell function, Bregs can potentially impair B cell effector-mediated anti-tumour response [95]. Finally, and of interest from the therapeutic point of view, CD39^+^CD73^+^ Bregs and their ability to produce extracellular adenosine are significantly decreased after chemotherapy [96]. Adding another level of complexity to the biology of Bregs, the immunoglobulin isotype expressed by B cells in the TME seems to be associated with their regulatory potential. Early in 2012, a research group in Japan described the presence of IgG4^+^ plasma cells in patients with cholangiocarcinoma, a rare form of hepatic cancer involving the bile ducts [97], The presence of the IgG4^+^ B cells was associated with increased frequency of CD4^+^Foxp3^+^ T cells and decreased CD8^+^ T cells, suggesting the evasion of immunosurveillance by CD8^+^ cytotoxic T cells. Further characterization of the regulatory mechanisms of IgG4^+^ B cells showed that in human colon cancer these cells are able to induce the development of M2-like macrophages that favour the induction of a tolerogenic environment that impairs anti-tumour responses, promotes tumour progression, and is associated with poor prognosis [98]. Another suppressive mechanism mediated by IgG4^+^ B cells, described in patients with oesophageal cancer and mouse models of breast, colon, and skin cancer, involves inhibition of ADCC [99]. It is possible that this suppressive mechanism involves the inhibition of IgG1 anti-tumour functions through binding and inhibition of FcγRI activation, as shown in a xenograft model of melanoma [100].

## Bregs in B cell malignancies?

As well as suppressing anti-tumour responses, B cells themselves can be the origin of transformed cells. Like solid tissue tumours, B cell lymphomas exploit immunoregulatory cells to protect themselves from antitumour responses. Analysis of mice in which B cells express a constitutively activated Notch-1 signalling pathway unveiled a mechanism in which B cells produce increased levels of IL-33 leading to induction of Tregs and Th2 responses, and downregulation of Th1 and CD8^+^ T cells activity [101]. IL-33, a member of the IL-1 superfamily, has pro-inflammatory properties and signalling through its receptor, ST2, has been implicated in the shaping of the Treg phenotype associated with gastric cancer [102]. Mirroring the results in the Notch-1 animal model, analysis of gene-expression by diffuse large B-cell lymphomas showed the presence of activated Notch-1 signalling which correlated with increased levels of IL-33 and Treg gene signatures [101]. CD24^+^CD38^+^ B cells in the bone marrow and peripheral blood of patients with multiple myeloma can inhibit the ADCC displayed by NK cells against myeloma cells. Interestingly, in this study evidence has been found of beneficial reciprocal interactions between myeloma cells and B cells. It has been shown that myeloma cells inhibit the apoptosis of B cells, thus allowing the maintenance of the immunosuppression that benefits myeloma progression [103]. The relevance of this B cell population has been highlighted using blocking anti-CD38 antibodies in chronic lymphocytic leukaemia (CLL) that targets and depletes both B and T regulatory cells. In this model, therapeutic anti-CD38 antibodies shift the balance from suppressive cells to reactive cells (Th17 and CD8+ T cells) that mediate an anti-CLL response [104]. It will be important to fully determine whether the responses seen are due to Bregs activity or these are part of the mechanism of survival displayed by actual malignant B cells.

When aggressive chemotherapy is required to control B cell malignancies, or other leukaemia or lymphomas, bone marrow transplantation can be required to re-establish the patient’s immune system. Graft versus host disease (GvHD) remains a severe, life-threatening, potential side effect of allogeneic hematopoietic stem cell transplantation (allo-HSCT) affecting around 50% of recipients worldwide [105]. Donor-derived B cells have been reported to contribute substantially to chronic GvHD following allo-HSCT [106, 107]. Elevated levels of CD19^+^CD21^low^ B cells in the peripheral blood of allo-HSCT recipients with acute leukaemia have been proposed as a biomarker of the development of GvHD in the lung [108], and rituximab mediated prophylactic pre-transplant B cell depletion reduces the incidence of GvHD in allo-HSCT recipients with CLL and mantle cell lymphoma [109]. However, regulatory B cells have also been suggested to be beneficial in an allo-HSCT setting by repressing GvHD without inhibiting anti-leukaemic immune responses.

Studies by Khoder *et al*. and de Masson *et al*. both identified a loss of IL-10-producing regulatory B cells in allo-HSCT recipients with GvHD compared to patients without GvHD [110, 111]. De Masson *et al*. also reported a loss of B cells with a previously described CD19^+^CD24^hi^CD27^+^ Breg phenotype [20] in patients with GvHD [111]. Both groups suggested that this loss of Breg function may contribute to the development of GvHD, and experimental mouse models of GvHD support this idea. For instance, adoptive transfer of donor derived CD5^+^CD1d^hi^ Bregs at the time of allogeneic bone marrow transplantation has been described to significantly reduce the severity of sclerodermatous GvHD [112], and in a murine leukaemia setting co-transfer of CD5^+^CD1d^hi^ Bregs could significantly reduce the severity of GvHD while importantly maintaining an effective immune response against murine acute myeloid leukaemia cells [113]. While cellular therapy with donor Bregs in humans may be distant, similar features may be present when cord blood is used as the source for haematopoietic stem cells for leukaemia/lymphoma patients; cord blood HSCT induces a robust expansion of IL-10 producing B cells that can suppress CD4^+^ T cell proliferation in those recipients that escape GvHD [114]. Thus, regulatory B cells may in fact be beneficial in HSCT treatment of patients with B cell malignancies.

## Breg localization in cancer

From the clinical and therapeutic points of view, it is important to determine where Bregs are localized, and how they promote cancer in different environments. In addition to their localization within primary tumours as part of the lymphocyte infiltrate, Bregs can also be found in tumour-draining lymph nodes (TDLN) and the peripheral blood. Recent research in mouse models of melanoma and breast cancer has shown that Bregs of a transitional 2-marginal zone (B220^+^IgM^hi^CD21^hi^CD23^+^) or a PD-1^-^PDL-1^+^ phenotype accumulate in TDLN just before the onset of metastasis [39, 115]. These results suggest that tumours induce the establishment of a tolerant microenvironment by inducing the accumulation of Bregs in TDLN. Metastatic tumour cells entering the lymphatic network might arrive to TDLN and encounter favourable, immune-suppressed conditions that would allow their dissemination to distal parts to establish secondary growths.

However, analyses of TDLN from patients with HNSCC have cast a doubt on whether Bregs have a suppressive role in that environment. Contrary to the general idea that Bregs have a pro-tumour effect, it has been shown that the frequency of Bregs (CD5^+^, CD1d^hi^CD5^+^, and CD24^hi^CD38^hi^) decreased as tumours progressed from early to late stages, and that the frequency of Bregs in TDLN was positively associated with good prognostic clinical parameters [116]. The authors also identified subsets of atypical memory B cells and although they do not provide functional or mechanistic information, their results suggest that a fine balance among the different B cell populations (stimulatory versus suppressive) in lymph nodes alters the metastatic potential of tumour cells in HNSCC. Studies in pre-clinical mouse models of pancreatic cancer have also challenged the general idea that Bregs favour tumour progression. Rather, the authors suggest that under certain conditions bulk B cell populations, which include Bregs, secrete immunostimulatory molecules and establish cell to cell interactions that contribute to anti-tumour responses [117]. Further evidence against a pro-tumour role for Bregs comes from study of patients with stage I breast cancer where there was no correlation between the frequency of Bregs in the peripheral blood and the tumour stage [39]. It would be important to determine in follow-up studies whether the frequency of Bregs at early stages of tumour development correlates with tumour aggressiveness and metastasis at more advanced stages [39].

As well as in the blood and TDLN, Bregs have been suggested to be present in tertiary lymphoid structures. For instance, analyses of hepatic lesions found in the early stages of liver carcinogenesis have shown enrichment of B cells and markers of immunosuppressive activity in TLS that might be associated with the full development of hepatocellular carcinoma [118].

The presence of stimulatory or suppressive B cells in different compartments of the TME might be determined by the pattern and expression level of adhesion molecules that allow the recruitment and homing of relevant immune cell populations. Analysis of gene expression in adenocarcinoma and squamous cell lung cancer has uncovered that overexpression of a set of endothelial adhesion molecule genes (including P-selectin, E-selectin, ICAM-1, VCAM-1, and ITGB1) favours infiltration of activated B cells (including Bregs) and Tregs versus the infiltration of effector CD4^+^ and cytotoxic CD8^+^ T cells. The opposite effect was seen when cellular barrier molecule genes (claudin-1/5/7, E-cadherin or desmoplakin) were overexpressed, in this case effector CD4^+^ and cytotoxic CD8^+^T cell infiltration was favoured over B cell infiltration [5]. Although no direct correlation between the expression of adhesion molecules with survival outcomes has been reported, this might be just one of multiple factors that determine the increased frequency and activity of Bregs and immunosuppressive T cell populations in TLS. Taken together, the apparent contradictory results on the suppressive role of Bregs might be influenced by the type and stage of cancer analysed. In the case of studies with animals some important facts have to be considered. Recruitment and infiltration of immune cells to primary tumours differ in quantity and cell immune types in genetic and injected orthotopic models. Also, studies with B-cell deficient mice [119] to evaluate the specific role of B cells in tumour biology have to be considered carefully as it might be possible that results are not a consequence simply of the lack of B cells but may be due to wider changes to the immune system of a mouse that develops in the absence of B cells [117].

## Therapeutic targeting of Bregs

In studies with potential therapeutic application, Bodogai *et al.*, have described a novel mechanism in which *in vivo* targeting of B cells with a conjugate of CXCL13 and CpG oligonucleotides (CXCL13/CpG-ODN) inhibits tBreg generation, resulting in blocking of cancer metastasis. CXCL13/CpG-ODN treatment induces expression of TNFR:TNF family member 4-1BBL (CD137L) on tBregs, which in turn elicits the activity of CD8^+^ cytotoxic T cells [120]. The use of small-molecule inhibitors to block Breg activity also has therapeutic potential. Cobimetinib and Tirabrutinib, inhibitors of the MAP kinase pathway and signalling molecule BTK, specifically inhibit IL-10 production and development of subsets CD1^hi^CD5^+^, TIM-1^+^ and CD21^+^CD23^+^CD24^+^Bregs without affecting other B cell subsets in mouse models of colon and pancreatic cancer [121, 122]. Targeting the inhibition or depletion of all B cells for therapeutic purposes in cancer remains controversial. Studies in renal cell carcinoma and melanoma have indicated that depletion of B cells with the monoclonal antibody rituximab (anti-CD20) does not have any effect on the clinical outcome of cancer [4]. Bodogai *et al* in contrast suggested that the use of rituximab depletes inflammatory B cells and actually enriches Bregs, as this subset expresses low levels of CD20, and that this might result in tumour progression [120]. However, a recently described strategy that specifically targets Bregs by use of a plasmid coding for a recombinant protein incorporating an anti-CD19 single-chain variable fragment and the extracellular domain of IL-10R1 (CD19scFV-IL-10R) appears to have had positive results. *In vivo* administration of this plasmid induced a significant reduction of Bregs and Tregs, enhanced cytotoxic CD8^+^T cells activity, and inhibited the development of hepatocellular carcinoma [33]. Current therapies used in oncology clinics could also have an impact on Bregs activity. Anti-PD-1 and anti-PD-L1 therapeutic antibodies have been successfully used as immune checkpoint inhibitors to treat some forms of cancer [123]. In view of the fact that PD-1/PD-L1 interactions are important for both Breg generation and function, it is tempting to suggest that antibodies that disrupt this interaction might also block the suppressive function of Bregs mediated by these two molecules. The description of critical molecules and mechanisms mediating the function of Bregs offers many possibilities for the development of inhibitors with potential therapeutic effects. The challenge for the development of therapeutic strategies will be to specifically target Bregs and disrupt interactions with other immune cells that lead to pro-tumour effects.

## Concluding remarks

Building up from extensive research in the infection and autoimmunity fields, different types of Bregs that promote tumour growth and metastasis have been described in many different animal models and types of cancer. A detailed description of the markers defining different Bregs, and the cell interactions and networks they establish with tumour-associated cells is critical for a full understanding of what triggers Bregs suppressive mechanisms and how they modulate anti-tumour immunity. This knowledge will allow the specific targeting and inhibition of Bregs activity that will result in effective cancer immunotherapies.

## Data Availability

Data sharing is not applicable to this article as no new data were created or analysed in this study.

## Abbreviations

ADCC: antibody-dependent cellular cytotoxicity
AMP: adenosine monophosphate
ATP: adenosine triphosphate
Breg: regulatory B cell
CLL: chronic lymphocytic leukaemia
GVHD: graft versus host disease
HNSCC: head and neck squamous cell carcinoma
HSCT: hematopoietic-stem cell transplantation
IL-10: interleukin-10
NK: natural killer
NSCLC: non-small cell lung cancer
TAMs: tumour-associated macrophages
TME: tumour microenvironment
TGF-β: transforming growth factor β
TDLN: tumour-draining lymph node
Treg: regulatory T cell
VEGF: vascular endothelial growth factor
